# Development and validation of a LASSO-based predictive model for inadvertent hypothermia in ICU patients

**DOI:** 10.3389/fmed.2025.1596030

**Published:** 2025-08-18

**Authors:** Xueting Wang, Yuxuan Chen, Lan Hua, Dongmei Wang, Xia Zhang, Lianhong Wang

**Affiliations:** ^1^Department of Critical Medicine, Affiliated Hospital of Zunyi Medical University, Zunyi, China; ^2^Nursing Department, Affiliated Hospital of Zunyi Medical University, Zunyi, China; ^3^Nursing College, Zunyi Medical University, Zunyi, China; ^4^Department of Critical Medicine, The Second Affiliated Hospital of Zunyi Medical University, Zunyi, China

**Keywords:** intensive care unit, critical patient, hypothermia, LASSO, predictive model

## Abstract

**Objective:**

To develop a risk predictive model for inadvertent hypothermia (IH) in intensive care unit (ICU) patients and to validate the accuracy of the model.

**Methods:**

The data was collected at the ICU of a tertiary hospital in Zunyi from November 2022 to June 2023 for model construction and internal validation. Data collected at the ICU of another tertiary hospital in Zunyi from July 2023 to December 2023 was used for external validation. The Least Absolute Shrinkage and Selection Operator (LASSO) was used to screen for strongly correlated predictors and build a predictive model, which was presented in the form of a nomogram and perform internal and external validation.

**Results:**

This study included a total of 720 participants, the incidence of IH in ICU patients was 18.19%. Six predictor variables were ultimately screened to construct the model: risk of IH in ICU patients = 1/(1 + exp−(−3.631 + 0.984 × catecholamines − 3.200 × antipyretic analgesics + 1.611 × RRT + 1.291 × invasive mechanical ventilation + 1.160 × GCS + 0.096 × lactate)). The results of the prediction model evaluation showed an AUC of 0.852 (95%*CI*: 0.805, 0.898) and internal validation yielded a C-statistic of 0.851. The Hosmer-Lemeshow test showed that *x^2^* = 7.438, *p* = 0.282 and the calibration curve showed that the actual prediction was close to the ideal prediction. The results of the DCA showed that the model is able to provide effective evidence to support clinical decision making. External validation showed an AUC of 0.846 (95%*CI*: 0.779, 0.913). The Hosmer-Lemeshow test showed *x^2^* = 13.041, *p* = 0.071 and the calibration curve was close to the ideal prediction situation.

**Conclusion:**

The IH predictive model for ICU patients constructed in this study passed both internal and external validation, and has good differentiation, calibration, clinical utility, and generalizability, which can help healthcare professionals to effectively identify high-risk groups for IH in the ICU.

## Introduction

Hypothermia is typically defined as a patient’s body temperature falling below 36 °C, resulting from excessive heat loss, reduced heat production, or impaired thermoregulation (1, 2). Hypothermia that occurs in patients, excluding cases of targeted temperature management, is often referred to as inadvertent hypothermia (IH) (3). IH is a common clinical abnormality in intensive care unit (ICU) patients, with an incidence ranging from 16 to 25% (1, 4, 5). IH can exacerbate or lead to severe adverse outcomes, including coagulation dysfunction, metabolic disturbances, elevated cardiovascular risk, organ dysfunction, immunosuppression, heightened infection risk, and delayed sedation recovery (6–10). Additionally, it contributes to prolonged hospital stays, increased healthcare costs, and poses a significant threat to patient safety (11). Studies have demonstrated that IH is an independent risk factor for ICU patient mortality, with a reported mortality rate ranging from 22 to 31% among ICU patients who develop IH (1, 12). Furthermore, mortality rates exhibit a positive correlation with both the severity of hypothermia and the prolonged duration of IH (3). Therefore, early identification of risk factors and stratification of high-risk populations are essential for preventing or mitigating the progression of IH.

Inadvertent hypothermia is potentially avoidable with early warning and management (13, 14). Some researchers have explored methods to identify patients at risk of hypothermia in the fields of perioperative, perinatal, emergency, and pediatric medicine (15–17). However, to our knowledge, no previous work has developed a model to predict hypothermia of ICU adult patients. Due to differences in disease types, severity, medications, and treatment modalities compared to perioperative, perinatal, emergency, and pediatric medicine, the risk factors for hypothermia in ICU patients may also differ. Existing hypothermia prediction models are not applicable to ICU patients. To prevent adverse reactions caused by inadvertent hypothermia, it is necessary to comprehensively identify risk factors for hypothermia in ICU patients and establish a prediction model.

As a result, our goal is to construct a hypothermia risk prediction model for ICU patients that enables early and accurate risk estimation. Considering that the risk factors affecting hypothermia in ICU patients may be numerous and complex, the key to constructing an accurate model lies in effectively extracting important independent variables and avoiding overfitting. We believe that the Least Absolute Shrinkage and Selection Operator (LASSO) can address these issues. LASSO, a machine learning method proposed by Tibshirani in 1996, controls model complexity through its constraint mechanism (18). LASSO selects the most relevant features for the final prediction model, which enhances its potential to perform well when applied to future patients (19). Therefore, this study aims to develop a hypothermia prediction model using LASSO and validate its performance. The success of this research can support clinical decision-makers in identifying high-risk patients and facilitate early interventions through improved clinical management.

## Method

### Participants

This was a prospective study. Adult ICU patients at the Affiliated Hospital of Zunyi Medical University were consecutively recruited between November 2022 and June 2023 as the development cohort and the internal validation cohort. Patients admitted to the adult ICU at the Second Affiliated Hospital of Zunyi Medical University from July 2023 to December 2023 were selected as the external validation cohort. Finally, patients who met all eligibility criteria and provided informed consent were included in the study. The inclusion criteria were as follows: (i) patients admitted to the ICU; (ii) age ≥ 18 years; (iii) ICU stay ≥ 48 h (to ensure effective data collection); and (iv) patients or their families willingly enrolled in the study. The exclusion criteria were as follows: (i) ICU readmission patients; (ii) patients with a body temperature < 36 °C before transfer to the ICU; and (iii) patients receiving targeted temperature management.

### Evaluation and measurement of IH

The temperature of the ICU ward was maintained at 22–24 °C, and the humidity was controlled at 50–60%. All patients were provided with a quilt upon admission, and covers were added or removed based on the patients’ needs and specific conditions.

In this study, axillary mercury thermometers were used to measure patient body temperature. After excluding cases undergoing targeted temperature management, axillary temperatures below 36.0 °C were defined as IH. The ICU nursing staff received uniform training in IH-related knowledge and temperature measurement procedures before the study began. Body temperature was measured and recorded regularly by the bedside nurses using axillary mercury thermometers at the time of admission, every 4 h thereafter, and immediately if abnormal body temperatures were suspected. To avoid false hypothermia readings due to issues such as displacement of the mercury thermometer caused by patient movement, the bedside nurse re-measured the temperature for patients with an initial reading below 36.0 °C and closely monitored the measurement process to confirm the accuracy of IH occurrence. Simultaneously, the condition of the hypothermic patient was reported to the physician for further treatment.

### Data acquisition

Through evidence-based research and expert discussions, we selected 36 candidate variables for the predictive model. These variables are all readily available in clinical settings, ensuring their practical utility for real-time applications. The candidate variables encompass general demographic factors, clinically relevant factors, and environmental factors. Specifically, they include: (1) data collected at admission, such as season, age, Body Mass Index (BMI), primary diagnosis, Sequential Organ Failure Assessment (SOFA), Acute Physiology and Chronic Health Evaluation (APACHE II), and assessed comorbidities (infection, shock, sepsis, hypertension, and chronic cardiovascular insufficiency); (2) data collected during ICU stay, including treatment status (e.g., surgery, renal replacement therapy (RRT), or invasive mechanical ventilation), physiological indicators [e.g., heart rate (HR), mean arterial pressure (MAP), and Glasgow Coma Score (GCS)], medication usage (e.g., sedatives, muscle relaxants, catecholamines, vasodilators, antipyretic analgesics, and glucocorticoids, plasma, red blood cells, platelets), the 24-h intravenous fluid intake from the previous day, and laboratory markers [e.g., pH, lactate, albumin, prealbumin, procalcitonin (PCT), white blood cell count, percentage of neutrophils, percentage of lymphocytes, and C-reactive Protein (CRP)]. Through expert evaluation, we recognized that critical laboratory parameters - including white blood cell count, neutrophil percentage, lymphocyte percentage, and pH values - exhibit clinically significant bidirectional fluctuations in ICU patients. These parameters may transition between subnormal ranges (indicating immunosuppressed states) and supranormal levels (reflecting inflammatory responses to infectious stressors) throughout a patient’s clinical course. Furthermore, pH extremes (acidosis or alkalosis) represent distinct pathophysiological mechanisms - including lactic acidosis, respiratory failure, or treatment-related effects - that may independently influence IH occurrence through impacts on tissue oxygenation and cellular metabolism. Given this dynamic variability of laboratory markers during critical illness, we systematically documented both peak and nadir values to fully characterize their clinical trajectories and potential associations with IH development. ECMO patients meeting inclusion criteria were enrolled and received standardized blood warming using heat exchanger in ECMO circuit. No IH events occurred in this subgroup, therefore ECMO treatment was not included as a potential influencing factor in the final analysis.

### Statistical analysis

Statistical analyses were performed using R language (version 4.3.2 for Windows) and SPSS 29.0. Normally distributed data were expressed as mean ± standard deviation (*x̄ ± s*) and analyzed using the independent samples t-test for comparisons between two groups. Non-normally distributed data were expressed as median and interquartile range *(M (Q1, Q3))* and analyzed using the Mann–Whitney U test. Categorical variables were expressed as numbers (percentages) (*n (%)*) and analyzed using the chi-square test or Fisher’s exact test.

Variables with >20% missing data were excluded, and the remaining missing values were imputed using random forest imputation. Continuous variables were then standardized to ensure uniform scaling for LASSO regression, which is sensitive to variable magnitude. This step prevents variables with larger units from dominating feature selection and facilitates algorithm convergence while enabling direct comparison of variable importance. The penalty parameter *λ* was determined through 10-fold cross-validation. This selected λ value was then applied in the LASSO regression equation to identify independent predictors with non-zero coefficients, thereby ensuring only relevant variables were retained in the final model.

A logistic regression model was constructed using the raw-scale (non-standardized) variables selected by LASSO to preserve clinical interpretability of odds ratios. Nomograms were developed to visualize the predictive model, which was internally validated using 1,000 bootstrap resamples. Discrimination was evaluated via the area under the receiver operating characteristic curve (AUC), while calibration was assessed using the Hosmer-Lemeshow test and calibration curves. Clinical utility was determined through decision curve analysis (DCA). External validation was performed using a spatio-temporal validation approach, leveraging data from an independent center collected during a different time period to ensure generalizability.

## Result

### Status of IH and patient characteristics

**Figure 1:**
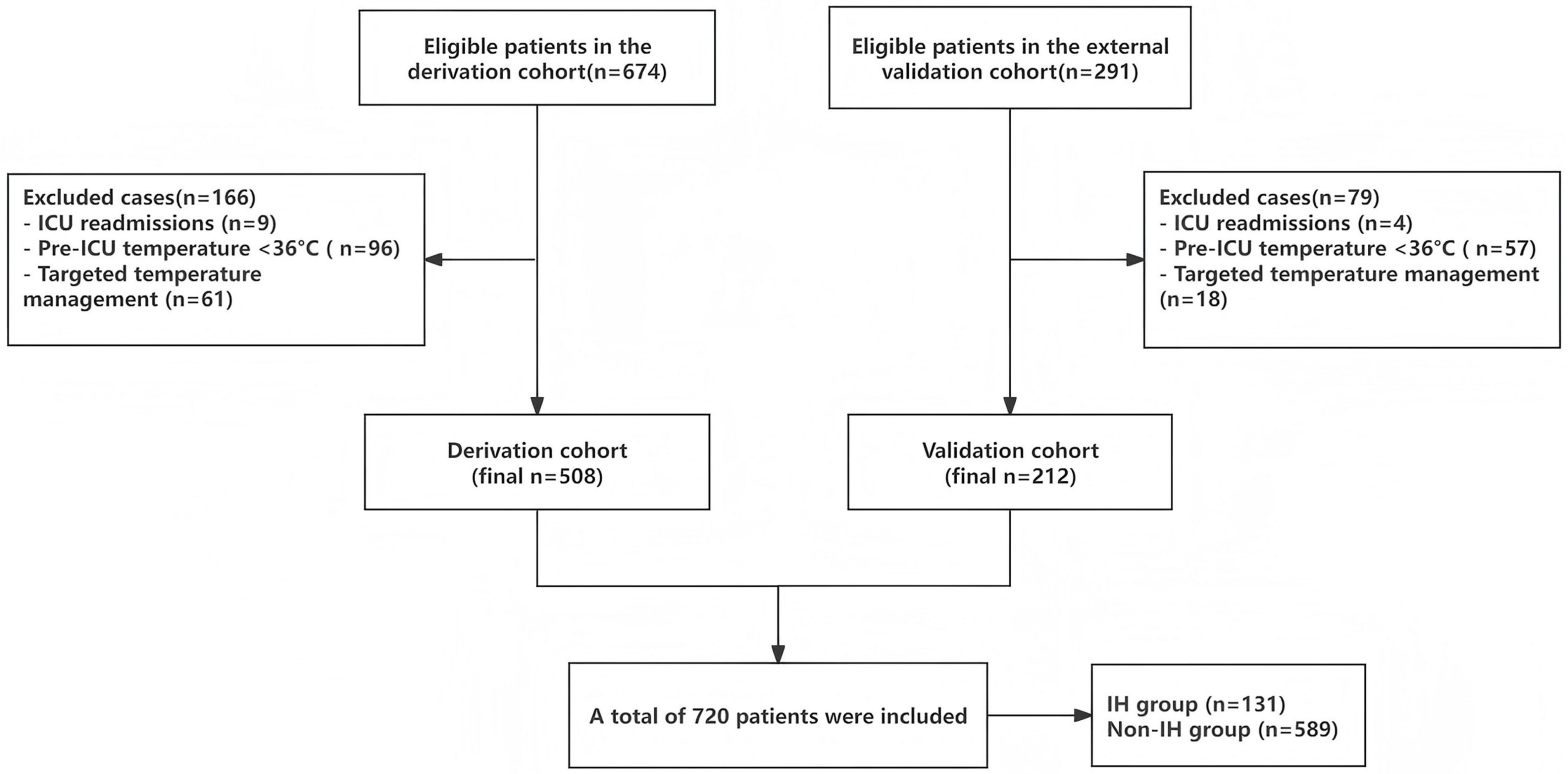
Flowchart of the patients included and analyzed in the study.

A final total of 720 study participants were included, comprising 589 non-IH patients and 131 IH patients, with an IH incidence of 18.19%. The derivation cohort included 508 cases (90 IH patients, 17.7%), and the external validation cohort included 212 cases (41 IH patients, 19.3%), Figure 1 shows the flow diagram of patient enrollment and analysis in the study. Table 1 presents the baseline characteristics of IH and non-IH groups in both cohorts.

**Table 1:** Comparative analysis of IH vs. non-IH groups in derivation and validation cohorts.

Variable	Derivation cohort (*n* = 508)				External Validation Cohort (*n* = 212)			
	IH group (*n* = 90)	Non-IH group (*n* = 418)	*χ^2^/t/z*	*p*	IH group (*n* = 55)	Non-IH group (*n* = 157)	*χ^2^/t/z*	*p*
*Primary Diagnosis, n (%)*			18.62	**0.017**			15.72	**0.47**
Circulatory System Diseases	7 (7.78)	30 (7.18)			6 (10.91)	11 (7.01)		
Respiratory System Diseases	20 (22.22)	97 (23.21)			18 (32.73)	48 (30.57)		
Nervous System Diseases	16 (17.78)	59 (14.11)			10 (18.18)	10 (6.37)		
Digestive System Diseases	13 (14.44)	93 (22.25)			9 (16.36)	33 (21.02)		
Endocrine, Nutritional, and Metabolic Diseases	1 (1.11)	15 (3.59)			0 (0.00)	3 (1.91)		
Genitourinary System Diseases	8 (8.89)	19 (4.55)			2 (3.64)	8 (5.10)		
Injuries, Poisonings, or External Causes	16 (17.78)	42 (10.05)			9 (16.36)	19 (12.10)		
Tumors	5 (5.56)	9 (2.15)			0 (0.00)	1 (0.64)		
Other System Diseases	4 (4.44)	54 (12.92)			1 (1.82)	24 (15.29)		
*Sepsis, n (%)*			6.79	**0.033**			2.46	0.292
Sepsis	4 (4.44)	22 (5.26)			1 (1.82)	9 (5.73)		
Septic Shock	26 (28.89)	71 (16.99)			15 (27.27)	31 (19.75)		
No Sepsis	60 (66.67)	325 (77.75)			39 (70.91)	117 (74.52)		
*Infection, n (%)*			0.04	0.848			0.12	0.730
Yes	44 (48.89)	209 (50.00)			29 (52.73)	87 (55.41)		
No	46 (51.11)	209 (50.00)			26 (47.27)	70 (44.59)		
*Shock, n (%)*			10.68	**0.001**			1.41	0.235
Yes	39 (43.33)	109 (26.08)			22 (40.00)	49 (31.21)		
No	51 (56.67)	309 (73.92)			33 (60.00)	108 (68.79)		
*Chronic Cardiovascular Insufficiency, n (%)*			0.41	0.520			0.09	0.761
Yes	16 (17.78)	63 (15.07)			13 (23.64)	34 (21.66)		
No	74 (82.22)	355 (84.93)			42 (76.36)	123 (78.34)		
*Hypertension, n (%)*			1.07	0.300			0.24	0.626
Yes	29 (32.22)	159 (38.04)			22 (40.00)	57 (36.31)		
No	61 (67.78)	259 (61.96)			33 (60.00)	100 (63.69)		
*Sedatives, n (%)*			2.61	0.106			3.46	0.063
Yes	56 (62.22)	221 (52.87)			40 (72.73)	92 (58.60)		
No	34 (37.78)	197 (47.13)			15 (27.27)	65 (41.40)		
*RRT, n (%)*			36.29	**<0.001**			7.21	**0.007**
Yes	36 (40.00)	55 (13.16)			19 (34.55)	27 (17.20)		
No	54 (60.00)	363 (86.84)			36 (65.45)	130 (82.80)		
*Invasive Mechanical Ventilation, n (%)*			16.30	**<0.001**			10.98	**<0.001**
Yes	80 (88.89)	283 (67.70)			50 (90.91)	107 (68.15)		
No	10 (11.11)	135 (32.30)			5 (9.09)	50 (31.85)		
*Surgery, n (%)*			3.384	0.496			11.795	**0.019**
Grade 1	0 (0.00)	3 (0.72)			1 (1.82)	0 (0.00)		
Grade 2	7 (7.78)	19 (4.55)			0 (0.00)	9 (5.73)		
Grade 3	9 (10.00)	61 (14.59)			3 (5.45)	25 (15.92)		
Grade 4	10 (11.11)	42 (10.05)			7 (12.73)	10 (6.37)		
No Surgery	64 (71.11)	293 (70.10)			44 (80.00)	113 (71.97)		
*GCS, n (%)*			23.22	**<0.001**			20.32	**<0.001**
≤8	33 (36.67)	62 (14.83)			22 (40.00)	19 (12.10)		
>8	57 (63.33)	356 (85.17)			33 (60.00)	138 (87.90)		
*Red Blood Cell Transfusion, n (%)*			1.45	0.228			1.07	0.300
Yes	30 (33.33)	113 (27.03)			21 (38.18)	48 (30.57)		
No	60 (66.67)	305 (72.97)			34 (61.82)	109 (69.43)		
*Platelet Transfusion, n (%)*			2.61	0.106			0.06	0.803
Yes	13 (14.44)	37 (8.85)			7 (12.73)	18 (11.46)		
No	77 (85.56)	381 (91.15)			48 (87.27)	139 (88.54)		
*Plasma Transfusion, n (%)*			12.05	**<0.001**			2.42	0.120
Yes	40 (44.44)	109 (26.08)			22 (40.00)	45 (28.66)		
No	50 (55.56)	309 (73.92)			33 (60.00)	112 (71.34)		
*Muscle Relaxants, n (%)*			6.12	**0.013**			6.66	**0.010**
Yes	9 (10.00)	14 (3.35)			9 (16.36)	7 (4.46)		
No	81 (90.00)	404 (96.65)			46 (83.64)	150 (95.54)		
*Catecholamines, n (%)*			26.29	**<0.001**			8.34	**0.004**
Yes	64 (71.11)	173 (41.39)			38 (69.09)	73 (46.50)		
No	26 (28.89)	245 (58.61)			17 (30.91)	84 (53.50)		
*Vasodilators, n (%)*			0.71	0.399			1.84	0.175
Yes	15 (16.67)	86 (20.57)			12 (21.82)	22 (14.01)		
No	75 (83.33)	332 (79.43)			43 (78.18)	135 (85.99)		
*Glucocorticoids, n (%)*			2.59	0.108			1.80	0.180
Yes	48 (53.33)	184 (44.02)			31 (56.36)	72 (45.86)		
No	42 (46.67)	234 (55.98)			24 (43.64)	85 (54.14)		
*Antipyretic Analgesics, n (%)*			29.54	**<0.001**			16.95	**<0.001**
Yes	3 (3.33)	130 (31.10)			2 (3.64)	49 (31.21)		
No	87 (96.67)	288 (68.90)			53 (96.36)	108 (68.79)		
*Season, n (%)*			–	–			–	–
Spring	35 (38.89)	168 (40.19)			–	–		
Summer	14 (15.56)	73 (17.46)			8 (14.55)	31 (19.75)		
Autumn	4 (4.44)	25 (5.98)			25 (45.45)	87 (55.41)		
Winter	37 (41.11)	152 (36.36)			22 (40.00)	39 (24.84)		
Age, mean ± SD	59.02 ± 17.85	57.59 ± 16.15	−0.75	0.454	58.09 ± 19.34	56.47 ± 17.47	−0.57	0.567
HR, mean ± SD	104.00 ± 26.08	102.76 ± 24.35	−0.43	0.666	100.00 ± 34.21	101.34 ± 20.78	0.27	0.785
MAP, mean ± SD	84.17 ± 21.87	90.26 ± 19.29	2.65	**0.008**	83.43 ± 25.68	88.71 ± 19.13	1.60	0.110
SOFA, mean ± SD	8.33 ± 4.00	5.93 ± 3.25	−5.33	**<0.001**	7.16 ± 3.59	5.99 ± 3.23	−2.26	**0.025**
24-h Intravenous Fluid Intake, mean ± SD	2894.73 ± 1560.75	2129.31 ± 1041.26	−4.44	**<0.001**	2662.93 ± 1414.78	2093.31 ± 1183.38	−2.92	**0.004**
Maximum pH, mean ± SD	7.46 ± 0.07	7.46 ± 0.07	−1.12	0.265	7.45 ± 0.08	7.45 ± 0.06	−0.13	0.894
Minimum pH, mean ± SD	7.30 ± 0.11	7.35 ± 0.08	3.85	**<0.001**	7.28 ± 0.13	7.33 ± 0.07	2.87	**0.005**
Albumin, mean ± SD	27.16 ± 5.15	28.60 ± 5.16	2.41	**0.016**	27.16 ± 4.06	28.51 ± 4.64	1.92	0.056
Prealbumin, mean ± SD	112.94 ± 64.26	117.26 ± 62.84	0.59	0.556	118.64 ± 62.09	124.35 ± 60.02	0.60	0.548
BMI, median (Q₁, Q₃)	22.03 (20.01, 24.00)	22.86 (20.61, 24.69)	−1.95	0.051	22.77 (20.28, 25.10)	22.68 (19.98, 24.80)	−0.46	0.643
APACHE II, median (Q₁, Q₃)	21.00 (14.00, 26.00)	17.00 (12.00, 21.00)	−3.70	**<0.001**	18.00 (14.00, 23.50)	16.00 (12.00, 20.00)	−2.49	**0.013**
Lactate, median (Q₁, Q₃)	2.80 (1.80, 4.38)	1.90 (1.40, 3.00)	−4.84	**<0.001**	3.20 (2.10, 6.25)	1.90 (1.40, 2.80)	−5.42	**<0.001**
PCT, median (Q₁, Q₃)	2.44 (0.58, 11.36)	1.31 (0.33, 6.23)	−2.54	**0.011**	2.01 (0.57, 18.50)	1.39 (0.31, 6.10)	−1.97	**0.049**
CRP, median (Q₁, Q₃)	150.55 (57.11, 184.25)	130.11 (56.55, 182.22)	−0.78	0.436	110.66 (56.09, 178.66)	105.19 (46.89, 178.38)	−0.52	0.602
Maximum White Blood Cell Count, median (Q₁, Q₃)	14.85 (10.53, 20.52)	13.63 (9.50, 17.94)	−1.92	0.054	15.14 (13.16, 21.48)	12.97 (9.81, 15.91)	−3.91	**<0.001**
Minimum White Blood Cell Count, median (Q₁, Q₃)	7.39 (5.84, 11.15)	7.72 (5.54, 10.33)	−0.39	0.695	7.41 (5.97, 11.54)	8.47 (5.75, 10.23)	−0.45	0.656
Maximum Neutrophil Percentage, median (Q₁, Q₃)	0.92 (0.88, 0.94)	0.88 (0.83, 0.93)	−4.42	**<0.001**	0.92 (0.86, 0.95)	0.89 (0.83, 0.92)	−3.37	**<0.001**
Minimum Neutrophil Percentage, median (Q₁, Q₃)	0.81 (0.74, 0.88)	0.78 (0.72, 0.84)	−2.89	**0.004**	0.84 (0.78, 0.89)	0.79 (0.74, 0.84)	−3.08	**0.002**
Maximum Lymphocyte Percentage, median (Q₁, Q₃)	0.04 (0.02, 0.06)	0.06 (0.03, 0.08)	−4.19	**<0.001**	0.04 (0.02, 0.06)	0.06 (0.04, 0.09)	−3.55	**<0.001**
Minimum Lymphocyte Percentage, median (Q₁, Q₃)	0.10 (0.06, 0.15)	0.12 (0.08, 0.17)	−2.39	**0.017**	0.09 (0.06, 0.12)	0.12 (0.08, 0.16)	−2.79	**0.005**

t, t-test; Z, Mann–Whitney test; χ^2^, Chi-square test; SD, standard deviation; M, Median; Q₁, 1st Quartile; Q₃, 3st Quartile.

Due to the influence of data collection timing, only a descriptive analysis of the seasons was conducted.Values in bold represent statistically significant results (*p* < 0.05).

To evaluate the model’s generalizability and identify potential biases, we compared baseline characteristics between the derivation and external validation cohorts. Most variables showed no statistically significant differences, except for chronic cardiovascular insufficiency and minimum pH (Table 2). The cohorts were generally comparable.

**Table 2:** Baseline characteristics of the derivation cohort and external validation cohort.

Variable	Derivation cohort (*n* = 508)	External validation cohort (*n* = 212)	*χ^2^/t/z*	*p*
*Primary Diagnosis, n (%)*			13.25	0.104
Circulatory System Diseases	37 (7.28)	17 (8.02)		
Respiratory System Diseases	117 (23.03)	66 (31.13)		
Nervous System Diseases	75 (14.76)	20 (9.43)		
Digestive System Diseases	106 (20.87)	42 (19.81)		
Endocrine, Nutritional, and Metabolic Diseases	16 (3.15)	3 (1.42)		
Genitourinary System Diseases	27 (5.31)	10 (4.72)		
Injuries, Poisonings, or External Causes	58 (11.42)	28 (13.21)		
Tumors	14 (2.76)	1 (0.47)		
Other System Diseases	58 (11.42)	25 (11.79)		
*Sepsis, n (%)*			0.66	0.721
Sepsis	26 (5.12)	10 (4.72)		
Septic Shock	97 (19.09)	46 (21.70)		
No Sepsis	385 (75.79)	156 (73.58)		
*Infection, n (%)*			1.45	0.229
Yes	253 (49.80)	116 (54.72)		
No	255 (50.20)	96 (45.28)		
*Shock, n (%)*			1.34	0.247
Yes	148 (29.13)	71 (33.49)		
No	360 (70.87)	141 (66.51)		
*Chronic Cardiovascular Insufficiency, n (%)*			4.54	0.033
Yes	79 (15.55)	47 (22.17)		
No	429 (84.45)	165 (77.83)		
*Hypertension, n (%)*			<0.01	0.948
Yes	188 (37.01)	79 (37.26)		
No	320 (62.99)	133 (62.74)		
*Sedatives, n (%)*			3.65	0.056
Yes	277 (54.53)	132 (62.26)		
No	231 (45.47)	80 (37.74)		
*RRT, n (%)*			1.39	0.238
Yes	91 (17.91)	46 (21.70)		
No	417 (82.09)	166 (78.30)		
*Invasive Mechanical Ventilation, n (%)*			0.50	0.478
Yes	363 (71.46)	157 (74.06)		
No	145 (28.54)	55 (25.94)		
*Surgery, n (%)*			1.38	0.848
Grade 1	3 (0.59)	1 (0.47)		
Grade 2	26 (5.12)	9 (4.25)		
Grade 3	70 (13.78)	28 (13.21)		
Grade 4	52 (10.24)	17 (8.02)		
No Surgery	357 (70.28)	157 (74.06)		
*GCS, n (%)*			<0.01	0.842
≤8	95 (18.70)	41 (19.34)		
>8	413 (81.30)	171 (80.66)		
*Red Blood Cell Transfusion, n (%)*			1.39	0.238
Yes	143 (28.15)	69 (32.55)		
No	365 (71.85)	143 (67.45)		
*Platelet Transfusion, n (%)*			0.61	0.435
Yes	50 (9.84)	25 (11.79)		
No	458 (90.16)	187 (88.21)		
*Plasma Transfusion, n (%)*			0.37	0.544
Yes	149 (29.33)	67 (31.60)		
No	359 (70.67)	145 (68.40)		
*Muscle Relaxants, n (%)*			2.66	0.103
Yes	23 (4.53)	16 (7.55)		
No	485 (95.47)	196 (92.45)		
*Catecholamines, n (%)*			1.95	0.163
Yes	237 (46.65)	111 (52.36)		
No	271 (53.35)	101 (47.64)		
*Vasodilators, n (%)*			1.45	0.228
Yes	101 (19.88)	34 (16.04)		
No	407 (80.12)	178 (83.96)		
*Glucocorticoids, n (%)*			0.51	0.475
Yes	232 (45.67)	103 (48.58)		
No	276 (54.33)	109 (51.42)		
*Antipyretic Analgesics, n (%)*			0.35	0.551
Yes	133 (26.18)	51 (24.06)		
No	375 (73.82)	161 (75.94)		
*Season, n (%)*			–	–
Spring	203 (39.96)	0 (0.00)		
Summer	87 (17.13)	39 (18.40)		
Autumn	29 (5.71)	112 (52.83)		
Winter	189 (37.20)	61 (28.77)		
Age, mean ± SD	57.84 ± 16.46	56.90 ± 17.94	0.68	0.494
HR, mean ± SD	102.98 ± 24.65	101.00 ± 24.89	0.98	0.326
MAP, mean ± SD	89.18 ± 19.89	87.34 ± 21.09	1.11	0.266
SOFA, mean ± SD	6.36 ± 3.51	5.97 ± 3.31	1.21	0.227
24-h Intravenous Fluid Intake, mean ± SD	2264.92 ± 1185.30	2241.08 ± 1268.96	0.24	0.810
Maximum pH, mean ± SD	7.45 ± 0.07	7.45 ± 0.06	1.62	0.106
Minimum pH, mean ± SD	7.33 ± 0.09	7.34 ± 0.09	2.86	0.004
Albumin, mean ± SD	28.35 ± 5.19	28.16 ± 4.53	0.45	0.655
Prealbumin, mean ± SD	116.50 ± 63.05	122.87 ± 60.47	−1.25	0.212
BMI, median (Q₁, Q₃)	22.70 (20.55, 24.62)	22.72 (20.13, 24.84)	−0.10	0.917
APACHE II, median (Q₁, Q₃)	17.50 (12.00, 22.00)	16.00 (12.00, 20.00)	−1.50	0.134
Lactate, median (Q₁, Q₃)	2.00 (1.40, 3.22)	2.10 (1.60, 3.40)	−1.08	0.280
PCT, median (Q₁, Q₃)	1.46 (0.34, 7.14)	1.50 (0.39, 7.35)	−0.50	0.614
CRP, median (Q₁, Q₃)	132.80 (56.63, 182.70)	108.97 (48.02, 178.88)	−1.78	0.074
Maximum White Blood Cell Count, median (Q₁, Q₃)	13.89 (9.73, 18.17)	13.49(10.29, 16.81)	−0.21	0.834
Minimum White Blood Cell Count, median (Q₁, Q₃)	7.67 (5.59, 10.41)	8.21(5.86, 10.38)	−1.03	0.301
Maximum Neutrophil Percentage, median (Q₁, Q₃)	0.89 (0.84, 0.93)	0.90 (0.84, 0.93)	−0.61	0.539
Minimum Neutrophil Percentage, median (Q₁, Q₃)	0.79 (0.72, 0.85)	0.80 (0.74, 0.86)	−1.36	0.173
Maximum Lymphocyte Percentage, median (Q₁, Q₃)	0.12 (0.08, 0.17)	0.11 (0.07, 0.16)	−1.01	0.314
Minimum Lymphocyte Percentage, median (Q₁, Q₃)	0.05 (0.03, 0.08)	0.05 (0.03, 0.08)	−0.32	0.747

t, t-test; Z, Mann–Whitney test; χ^2^, Chi-square test; SD, standard deviation; M, Median; Q₁, 1st Quartile; Q₃, 3st Quartile.

Due to the influence of data collection timing, the number of patients collected in the two centers was not comparable across different seasons. Therefore, only a descriptive analysis of the seasons was conducted.

### Variable selection

**Figure 2:**
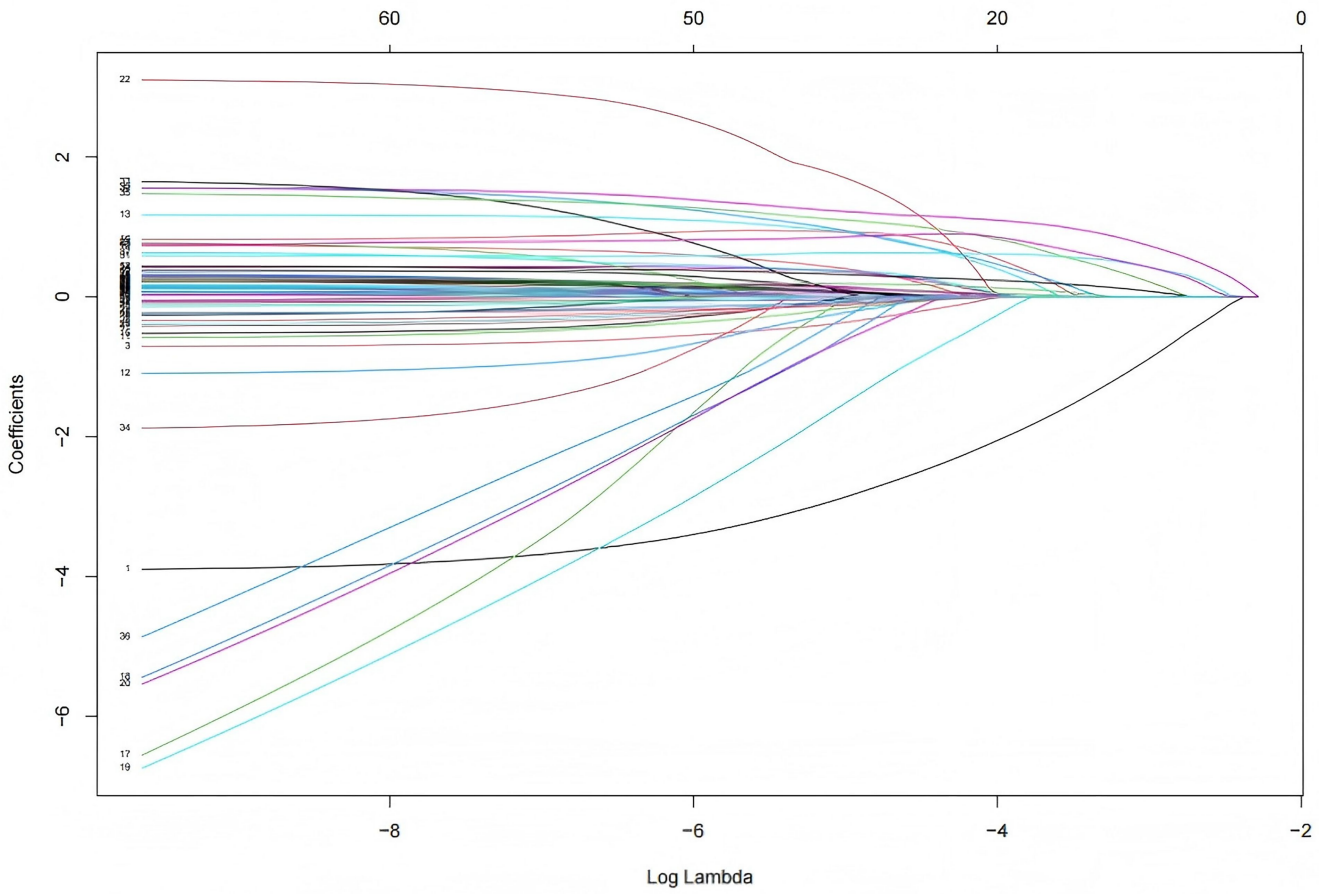
Coefficient path of LASSO regression. Each colored line represents the coefficient trajectory of an independent variable as the penalty parameter *λ* changes. The x-axis shows log (λ), indicating the strength of penalization, while the y-axis displays the magnitude of regression coefficients. As λ increases, the coefficients shrink toward zero, indicating variable exclusion. As λ decreases, some coefficients grow and stabilize.

**Figure 3:**
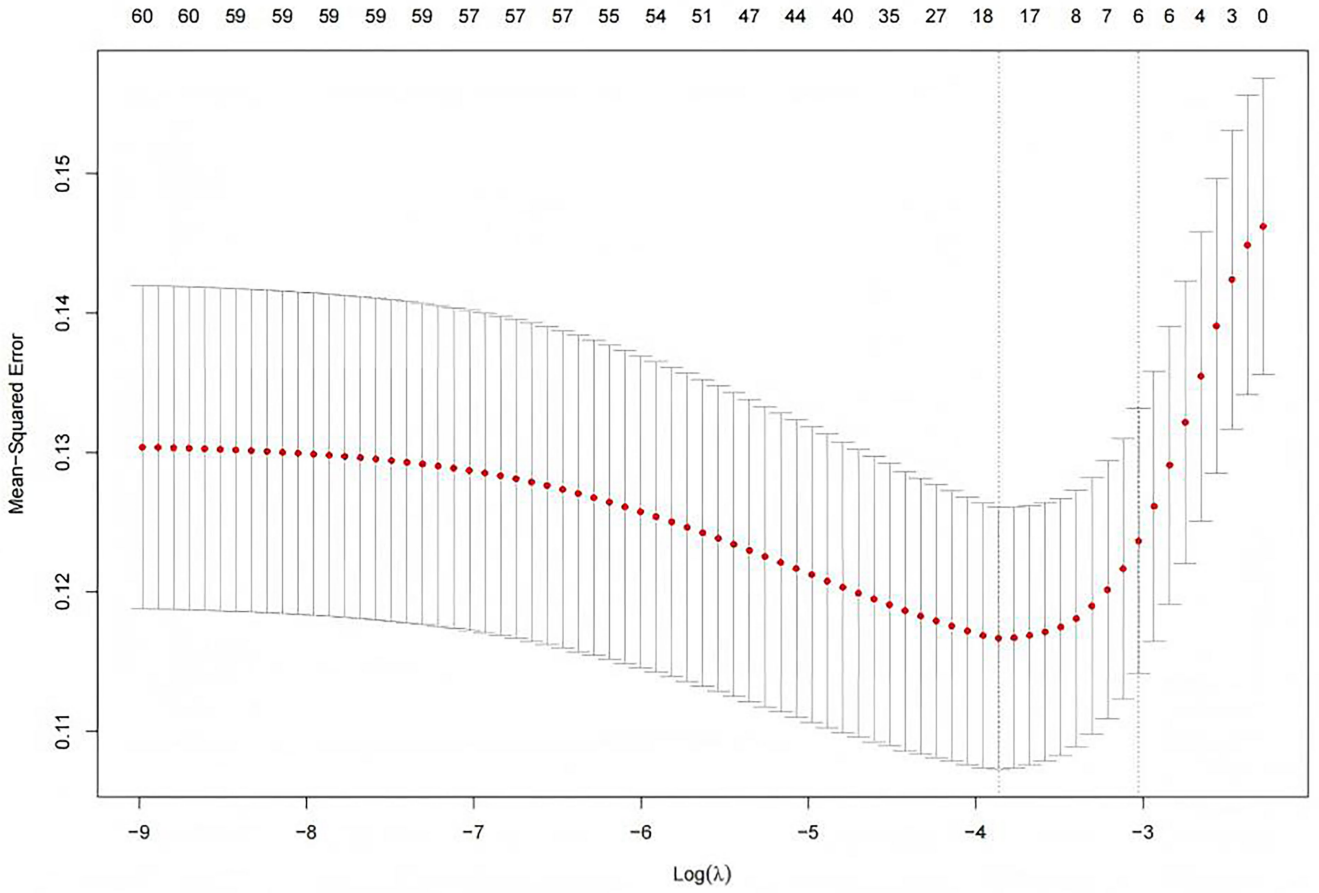
Selection of the penalty parameter λ using ten fold cross validation. Figure shows two λ selection criteria in LASSO regression. The left dashed line (λ-min = 0.0191) represents the value with minimal cross-validation error (optimal predictive accuracy), selecting 18 variables for higher complexity. The right line (λ-1se = 0.0485) follows the one-standard-error rule, choosing the simplest model within 1 SE of the minimum error and retaining only 6 variables.

In the original data, the missing values for PCT accounted for 6.69%. The random forest method was employed for missing value imputation, and the results indicated that the imputed data closely approximated the original data. Finally, continuous variables were standardized using the Z-score normalization method. The occurrence of IH in ICU patients (yes/no) was defined as the dependent variable, and the normalized data were incorporated into the LASSO regression model. As illustrated in Figure 2, the regression coefficients of the independent variables were progressively shrunk toward zero with increasing penalty coefficient *λ*. Figure 3 displays 10-fold cross-validation results. The left dashed line (λ-min) represents the λ value minimizing prediction error (18 variables), while the right line (λ-1se) selects the most parsimonious model within 1 SE of minimum error (6 variables). We selected λ-1se (0.0485) to optimize clinical utility through balanced accuracy and simplicity. Ultimately, six predictors were identified: catecholamine, RRT, invasive mechanical ventilation, lactate, GCS, and antipyretic analgesic.

### Model development

The prediction model was constructed by incorporating the six selected predictors into a logistic regression model. The formula of the model is presented as follows: Risk of IH in ICU patients = 1 /(1 + exp. − (− 3.631 + 0.984 × catecholamine − 3.200 × antipyretic analgesics + 1.611 × RRT + 1.291 × invasive mechanical ventilation + 1.160 × GCS + 0.096 × lactate)). Table 3 presents the results of the logistic regression analysis of factors influencing IH in ICU patients.

**Table 3:** Results of logistic regression analysis of factors influencing IH in ICU patients.

Variable	*β*	*SE*	*p*	*OR*	*95% CI*
Intercept	−3.631	0.407	<0.001	0.026	(0.012, 0.059)
Catecholamines	0.984	0.301	0.001	2.674	(1.483, 4.821)
Antipyretic analgesics	−3.200	0.623	<0.001	0.041	(0.012, 0.138)
RRT	1.611	0.323	<0.001	5.007	(2.659, 9.429)
Invasive mechanical ventilation	1.291	0.398	0.001	3.636	(1.666, 7.935)
GCS	1.160	0.317	<0.001	3.190	(1.714, 5.939)
Lactate	0.096	0.054	0.075	1.101	(0.990, 1.224)

β, regression coefficient; SE, standard error; OR, odds ratio; CI, confidence interval; RRT, Renal Replacement Therapy; GCS, Glasgow Coma Score.

**Figure 4:**
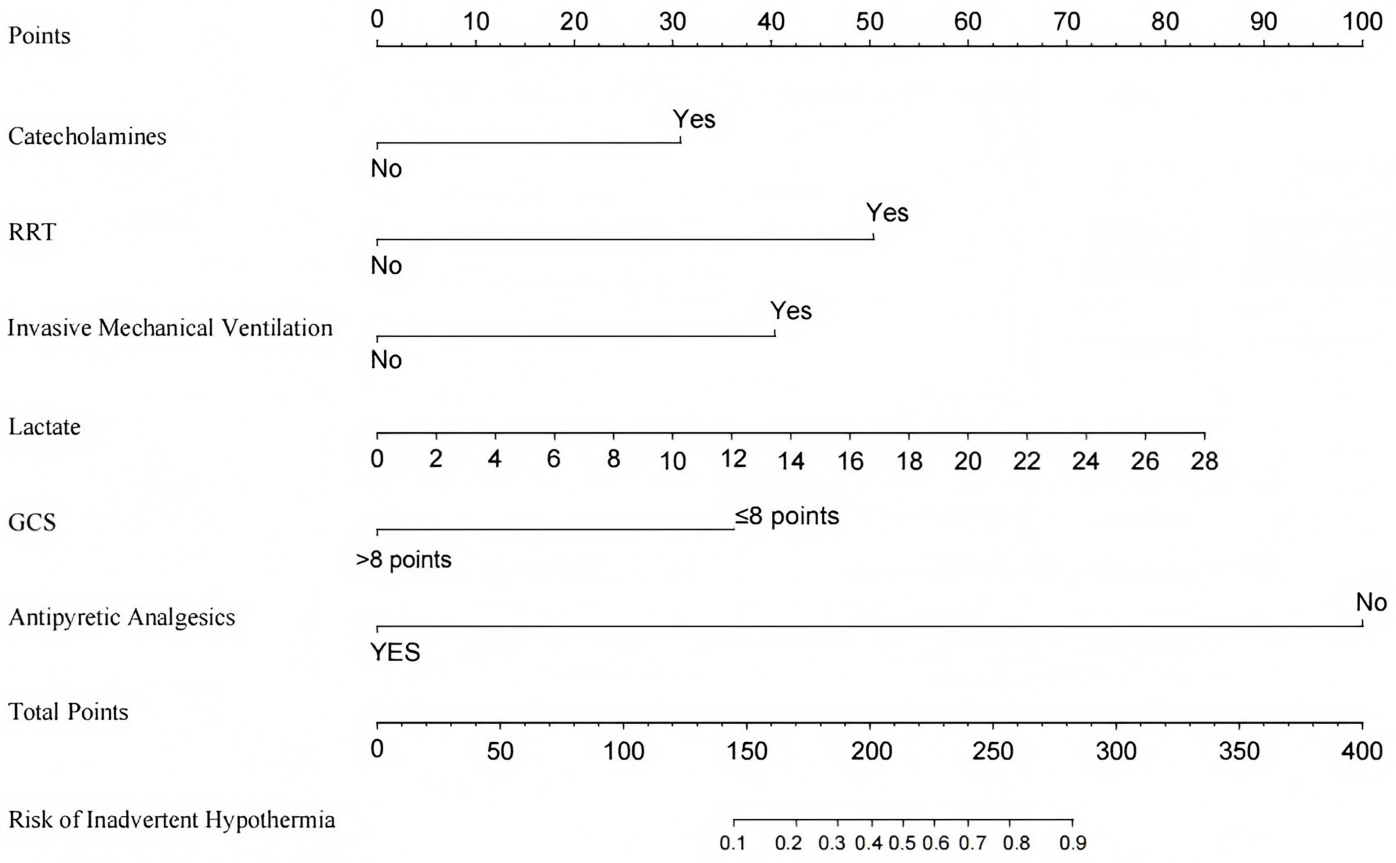
Nomogram for predicting the risk of IH in ICU patients. RRT, Renal Replacement Therapy; GCS, Glasgow Coma Score.

The constructed prediction model was visualized as a risk nomogram. The nomogram projects the original measurements or categorical outcomes of the six independent variables in the model onto the top point scale line to obtain corresponding points. These points are then summed to yield a total score, which is projected downward onto the bottom axis to predict the probability of IH occurrence in ICU patients, as shown in Figure 4.

### Evaluation and internal validation

**Figure 5:**
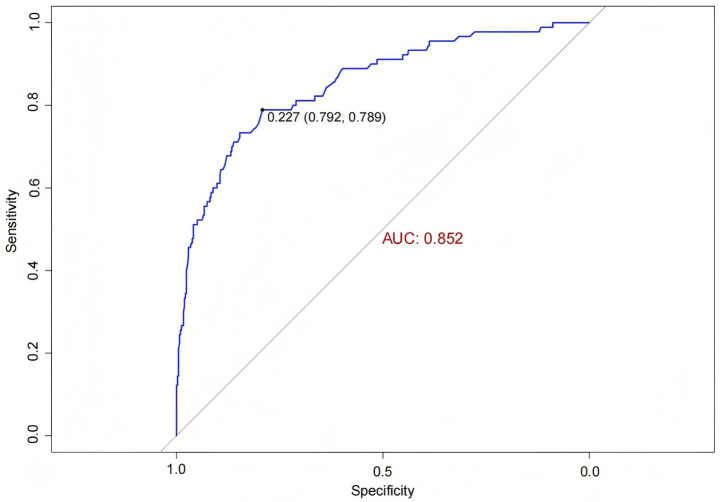
ROC curve of the IH prediction model in ICU patients.

ROC curve analysis was used to evaluate the predictive ability of the model for IH, yielding an AUC of 0.852 (95% *CI*: 0.805, 0.898). The optimal cutoff value on the ROC curve was 0.227, demonstrating good specificity and sensitivity of 79.2 and 78.9%, respectively (Figure 5). Internal validation using bootstrap resampling with 1,000 iterations resulted in a C-statistic of 0.851, indicating good discriminative ability of the model.

**Figure 6:**
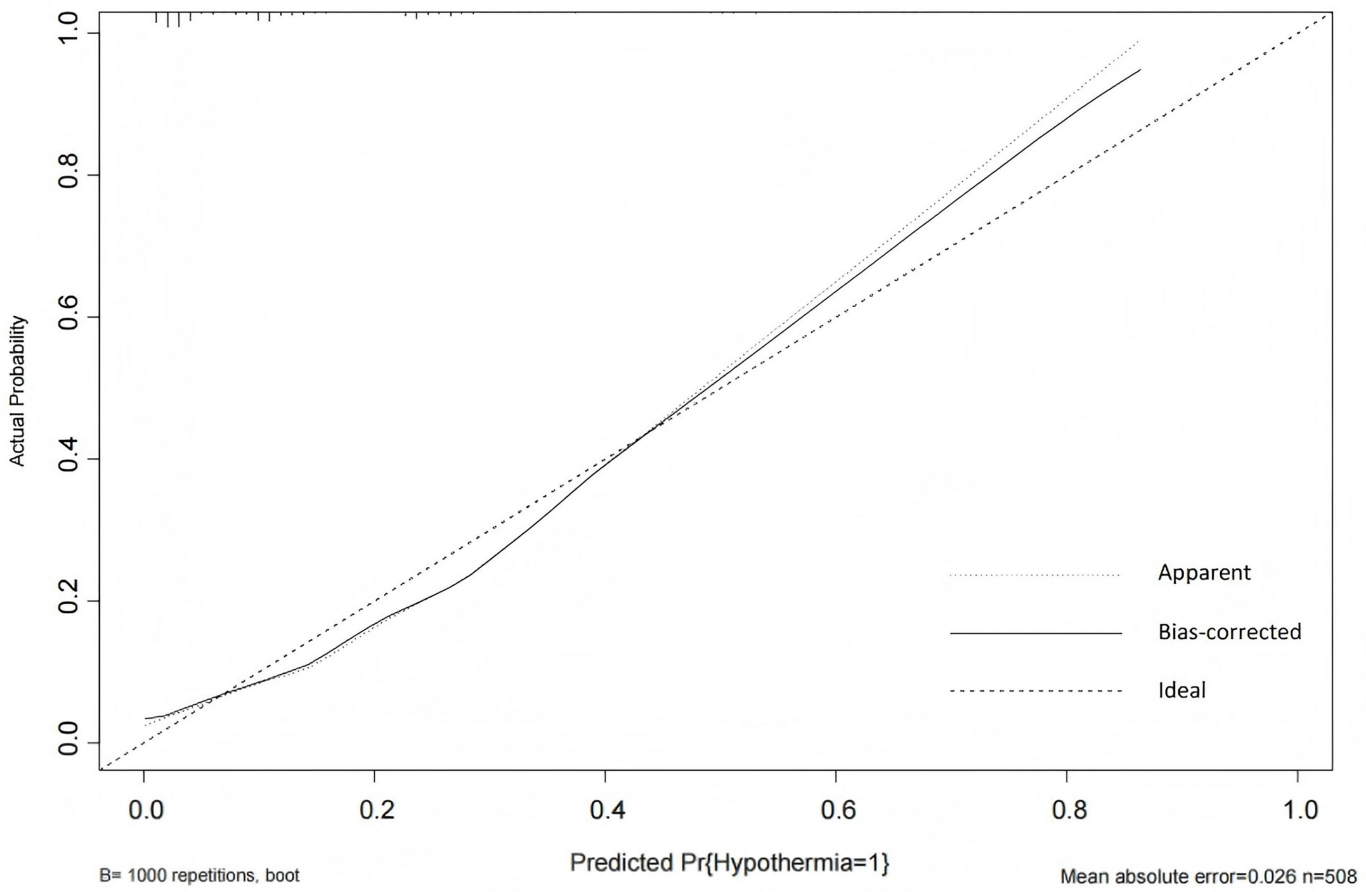
Calibration curve of the IH prediction model in ICU patients. The calibration plot includes three reference lines: (1) the Ideal Line, representing perfect agreement between predicted and observed probabilities, which serves as the calibration benchmark; (2) the Apparent Line, showing the actual relationship between model predictions and observed event rates (closer proximity to the Ideal Line indicates better accuracy); and (3) the Bias-Corrected Line, derived through bootstrap resampling to adjust for overoptimism and estimate the model’s stable performance in external populations.

The Hosmer-Lemeshow goodness-of-fit test yielded a *χ^2^* value of 7.438 with a *p*-value of 0.282 (>0.05), indicating good model fit. The calibration curve (Figure 6) showed that both the predicted curve and the corrected calibration curve were close to the ideal curve, demonstrating good agreement between the predicted and actual probabilities of IH occurrence in ICU patients.

**Figure 7:**
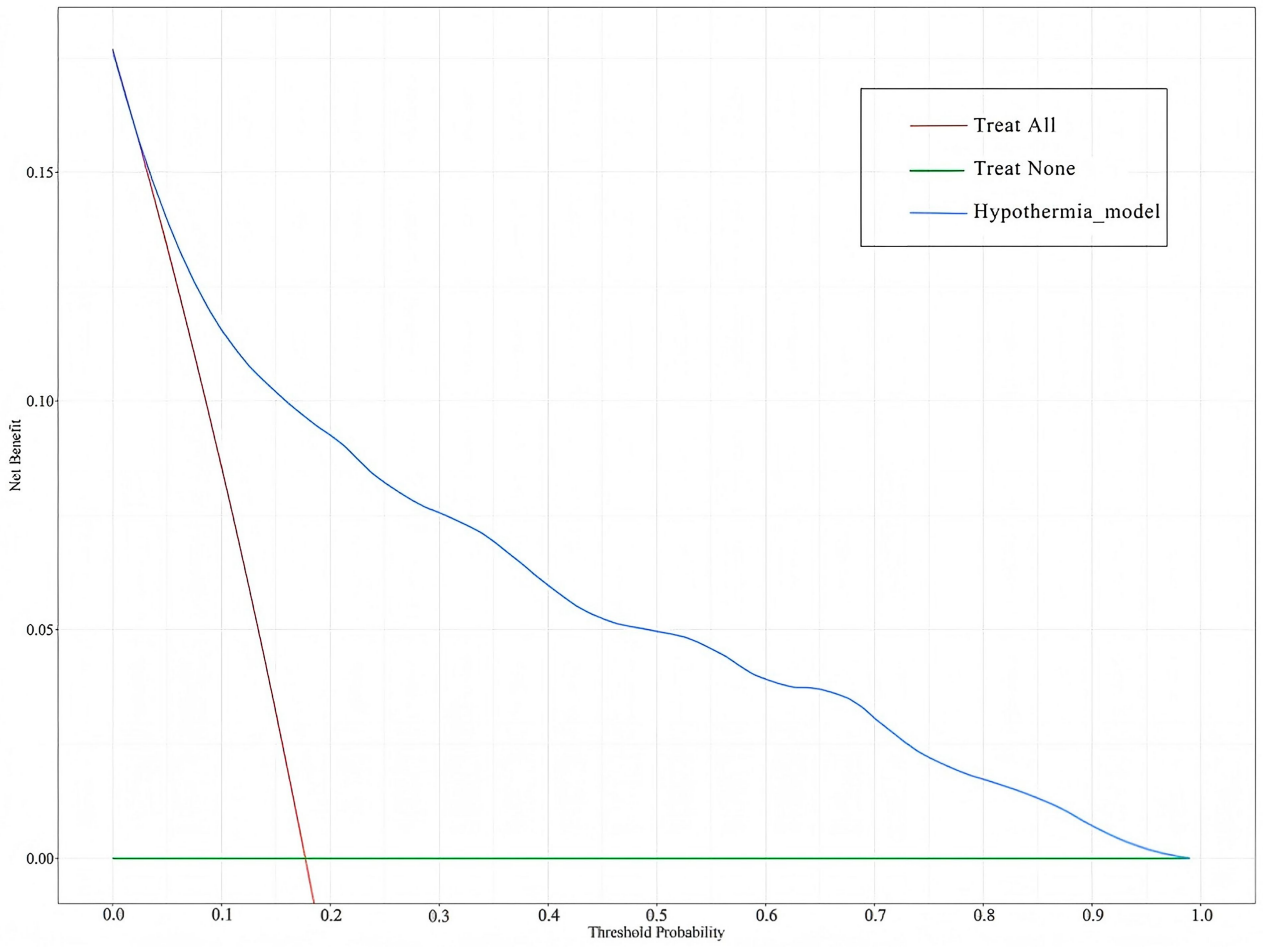
DCA of the IH prediction model in ICU patients. The green horizontal line assumes that no ICU patients developed IH and none received intervention, while the red diagonal line indicates that all patients developed IH and all received intervention. The blue curve represents the net benefit of the IH prediction model.

The clinical utility of the prediction model was evaluated using decision curve analysis (DCA). As shown in Figure 7, the green horizontal line assumes that no ICU patients developed IH and none received intervention, while the red diagonal line indicates that all patients developed IH and all received intervention. The blue curve represents the net benefit of the IH prediction model. A greater distance between the blue curve and the two reference lines (green and red) corresponds to higher net benefit and clinical value. Within the threshold probability range of 0.04–0.98, the nomogram model demonstrated good clinical utility.

### External validation

**Figure 8:**
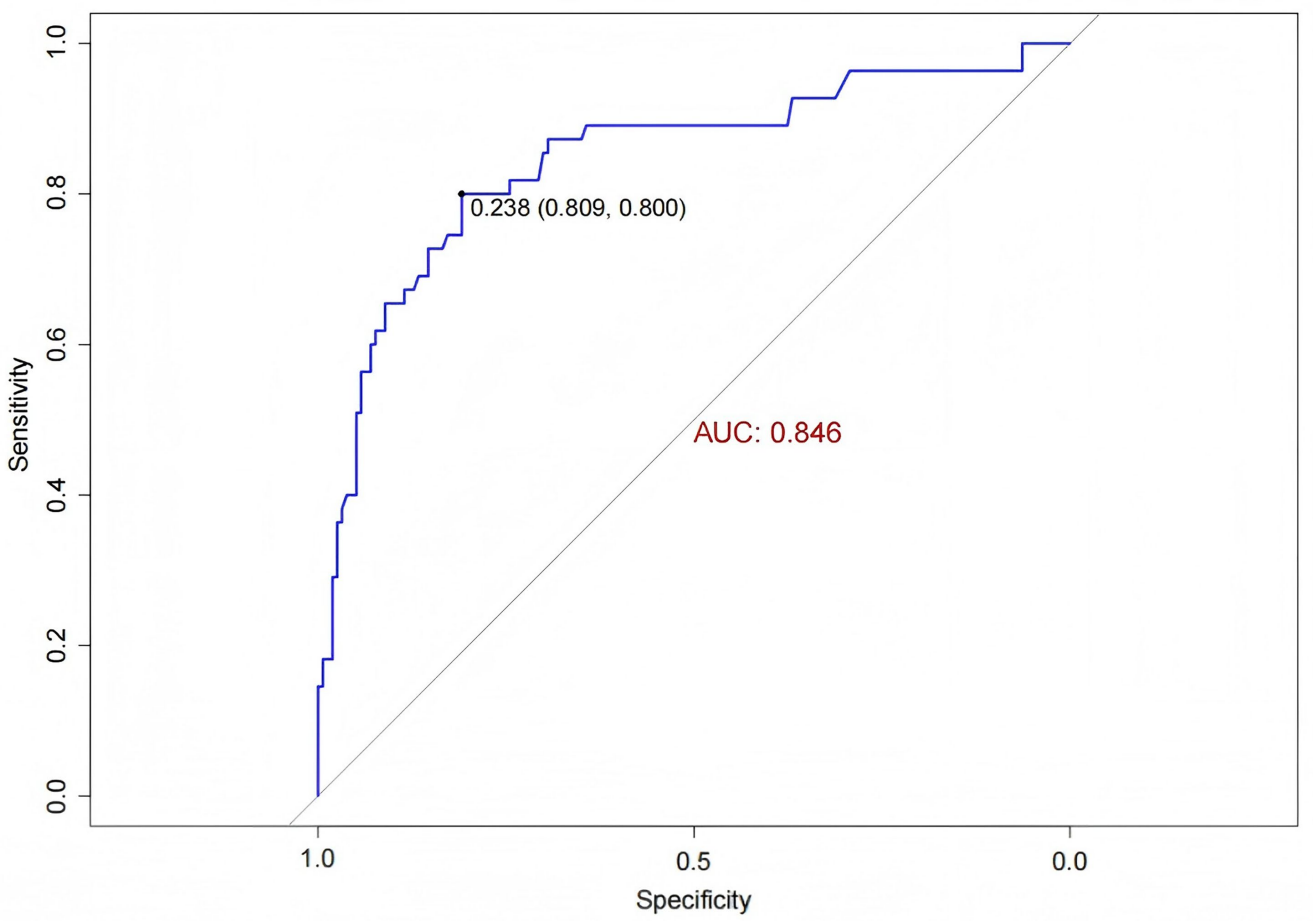
ROC curve for external validation of the IH prediction model in ICU patients.

The external validation data were obtained from the general ICU of another center. ROC curve analysis was performed to evaluate the predictive ability of the model for IH risk in the external validation cohort, yielding an AUC of 0.846 (95% *CI*: 0.779, 0.913) (Figure 8). The IH prediction model for ICU patients demonstrated good discriminative ability in the external validation.

**Figure 9:**
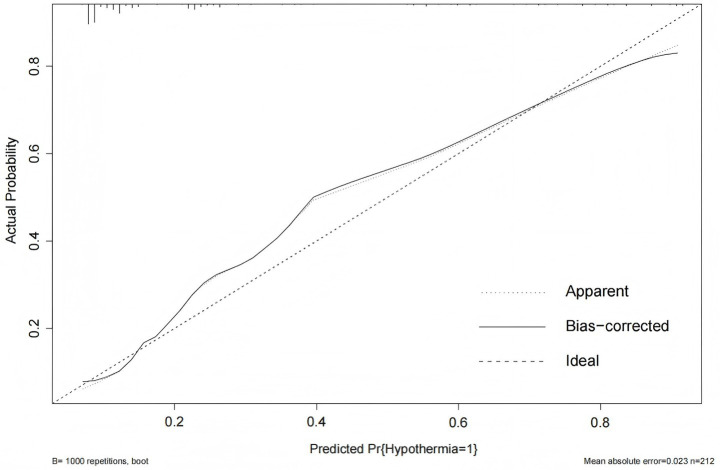
Calibration curve for external validation of the IH prediction model in ICU patients.

The Hosmer-Lemeshow goodness-of-fit test for the external validation of the prediction model yielded a *χ^2^* value of 13.041 with a *p*-value of 0.071 (>0.05). The calibration curve demonstrated that the predicted probabilities of IH risk in the external validation cohort were close to the actual observed probabilities (Figure 9), indicating good calibration of the IH prediction model for ICU patients.

## Discussion

ICU patients are critically ill with complex and rapidly changing conditions, and the occurrence of IH may further exacerbate clinical deterioration and complicate treatment (20). In the context of the widespread adoption of closed management in ICUs in China, healthcare providers, as the core of risk control, urgently need to improve their ability to early identify IH risks and implement targeted interventions to enhance patient outcomes, improve quality of life, and alleviate the burden on families and society. However, the current lack of effective tools for early identification of high-risk IH populations in ICUs may, to some extent, negatively impact patient clinical outcomes. Therefore, based on evidence-based methods and expert consensus, this study systematically integrated IH-related influencing factors and utilized the advantage of LASSO regression for automatic feature selection to construct a risk prediction model for IH in ICU patients, achieving early and accurate identification of high-risk IH populations. To improve the clinical utility of the model, the complex prediction formula was converted into an intuitive nomogram. The nomogram visually presents the contribution of each predictor, enabling clinicians to quickly obtain relevant indicators through bedside assessment or electronic medical records, thereby calculating individualized IH risk probabilities for patients (21). Moreover, the predictors incorporated into the nomogram in this study are routinely monitored in the intensive care unit setting, making them readily accessible and highly applicable in clinical practice.

The model included six predictors: catecholamines, RRT, invasive mechanical ventilation, lactate, GCS, and antipyretic analgesics. Among these, RRT, invasive mechanical ventilation, lactate, and GCS have been widely recognized as independent risk factors for IH in multiple studies (1, 22, 23), while the effects of catecholamines and antipyretic analgesics on IH have not been extensively studied or reported in previous literature. This study revealed that the use of catecholamines was significantly associated with an increased risk of IH, potentially due to their complex effects on hemodynamics and thermoregulation. While catecholamines reduce heat loss by constricting peripheral blood vessels, they may simultaneously exacerbate inadequate tissue perfusion and diminish heat production (24). Moreover, patients receiving catecholamines are typically in more critical conditions, often accompanied by metabolic disturbances and organ dysfunction, factors that inherently elevate the risk of hypothermia (25). Future studies should further explore the impact of the types, dosages, and duration of catecholamines use on hypothermia, while accounting for potential confounding variables. Interestingly, this study also found that the use of antipyretic analgesics was associated with a reduced risk of IH, a finding that contradicts initial expectations. Potential explanations include the anti-inflammatory effects of antipyretic analgesics mitigating inflammatory responses, or the possibility that these drugs are preferentially administered to febrile patients (26). Further research is warranted to elucidate the underlying mechanisms and address potential confounding factors.

Model performance validation is a critical step to ensure reliability and generalizability (27). This study comprehensively evaluated the model’s performance through both internal and external validation. Internal validation, conducted using the Bootstrap method with 1,000 repetitions, yielded a C-statistic of 0.851, indicating good internal consistency and stability while mitigating the risk of overfitting. For external validation, a temporal–spatial validation approach was employed, testing the model’s performance on new datasets from different centers and time points. This resulted in an AUC of 0.846 (95% *CI*: 0.779, 0.913), demonstrating satisfactory discriminative ability. Additionally, the Hosmer-Lemeshow goodness-of-fit test produced a *p*-value of 0.071, and the calibration curve closely aligned with the ideal curve, further supporting the model’s good calibration and consistency. Taken together, the internal and external validation results indicate that the model exhibits good discriminative ability, calibration, and generalizability, making it suitable for clinical application.

Nevertheless, this study has some limitations. First, this study did not assess the impact of massive transfusion on body temperature, potentially underestimating its contribution to hypothermia development. Second, the mechanisms underlying certain predictors (e.g., catecholamines and antipyretics) remain incompletely understood and require further investigation. Moreover, the model’s real-time dynamic prediction capability has not been evaluated. Future studies could explore integrating the model with continuous monitoring data to enhance its timeliness and clinical utility. Additionally, incorporating advanced intelligent technologies to link the model with real-time physiological monitoring data may enable dynamic risk prediction and early warning of IH, further improving its practical application. Finally, our findings may not be generalizable to Patients with abdominal compartment and abdomen apertum, as such cases were absent from our cohort due to our center’s treatment protocols. Future multicenter studies should validate the impact on IH in these high-risk subgroups.

## Conclusion

The influencing factors of IH in ICU patients are multifaceted, with catecholamines, RRT, invasive mechanical ventilation, lactate levels, GCS scores, and antipyretic analgesics identified as independent predictors. Therefore, the prevention of IH in ICU patients requires a comprehensive and multidisciplinary approach. The early warning model for IH developed in this study demonstrates good discriminative ability, calibration, clinical utility, and generalizability. It can assist healthcare providers in effectively identifying high-risk patients at an early stage, facilitating timely interventions and ultimately improving patient outcomes.

## Data Availability

The raw data supporting the conclusions of this article will be made available by the authors, without undue reservation.
