# Supplementary material for: Development and validation of a LASSO-based predictive model for inadvertent hypothermia in ICU patients
Source: Front Med (Lausanne). 2025 Aug 18;12:1596030. doi: 10.3389/fmed.2025.1596030 (PMC12399657; doi:10.3389/fmed.2025.1596030)
Supplement: Supplementary file 4 [file Data_Sheet_4.docx]

# Load the necessary packages

library(glmnet)

library(caret)

library(rms)

library(pROC)

library(ggplot2)

# LASSO

x <- as.matrix(df_normalized[, -which(names(df_normalized) == "y")])

y <- df_normalized$y

# Ten-fold cross-validation selection lambda

set.seed(123)

cv_fit <- cv.glmnet(x, y, alpha = 1, family = "binomial", type.measure = "auc", nfolds = 10)

# Plotting LASSO coefficient paths

plot(cv_fit$glmnet.fit, xvar = "lambda", label = TRUE)

title("LASSO Coefficient Path")

# Plotting lambda selection graphs for ten-fold cross-validation

plot(cv_fit)

title("Cross-Validation for Lambda Selection")

# Selection of the optimal lambda

optimal_lambda <- cv_fit$lambda.min

# Fitting LASSO models using optimal lambda

lasso_model <- glmnet(x, y, alpha = 1, family = "binomial", lambda = optimal_lambda)

# Extraction of filtered variables

selected_vars <- which(coef(lasso_model) != 0)

selected_vars_names <- rownames(coef(lasso_model))[selected_vars]

print("Selected Variables:")

print(selected_vars_names)

#Constructing a logistic regression model

df_selected <- df_normalized[, c("y", selected_vars_names)]

logistic_model <- glm(y ~ ., data = df_selected, family = binomial)

# Plotting columns and lines

ddist <- datadist(df_selected)

options(datadist = "ddist")

nomogram <- nomogram(logistic_model, fun = plogis, funlabel = "Risk Probability")

plot(nomogram)

# Model Evaluation - ROC Curve

pred_prob <- predict(logistic_model, type = "response")

roc_curve <- roc(y, pred_prob)

plot(roc_curve, main = "ROC Curve")

auc(roc_curve)

# Internal Validation - Bootstrap

set.seed(123)

boot_validate <- validate(logistic_model, method = "boot", B = 1000)

print(boot_validate)

# Plotting calibration curves

cal_curve <- calibrate(logistic_model, method = "boot", B = 1000)

plot(cal_curve, main = "Calibration Curve")

# Clinical Decision Curve

dca_curve <- dca(logistic_model, data = df_selected)

plot(dca_curve, main = "Decision Curve Analysis")

# external validation

df_external_normalized <- predict(preprocess_params, df_external)

# Forecasting external data

external_pred_prob <- predict(logistic_model, newdata = df_external_normalized, type = "response")

external_roc_curve <- roc(df_external_normalized$y, external_pred_prob)

plot(external_roc_curve, main = "External Validation ROC Curve")

auc(external_roc_curve)

# Externally verified calibration curves

external_cal_curve <- calibrate(logistic_model, newdata = df_external_normalized, method = "boot", B = 200)

plot(external_cal_curve, main = "External Validation Calibration Curve")
